# Orthodontic Treatment Need and Complexity among Nigerian Adolescents in Rivers State, Nigeria

**DOI:** 10.1155/2011/813525

**Published:** 2011-10-30

**Authors:** Elfleda Angelina Aikins, Oluranti Olatokunbo daCosta, Chukwudi Ochi Onyeaso, Michael Chukwudi Isiekwe

**Affiliations:** ^1^Department of Child Dental Health, Dental Centre, University of Port Harcourt Teaching Hospital, Port Harcourt 500001, Nigeria; ^2^Department of Child Dental Health, School of Dentistry, College of Medicine, University of Lagos, Lagos 101241, Nigeria; ^3^Department of Child Dental Health, Faculty of Dentistry, College of Health Sciences, University of Port Harcourt, Port Harcourt 500001, Nigeria

## Abstract

*Introduction*. The assessment of orthodontic treatment need and complexity are necessary for informed planning of orthodontic services. The aim of this cross-sectional study was to assess these parameters using the Index of Complexity, Outcome, and Need (ICON) in a Nigerian adolescent population in a region where orthodontic services are just being established. *Methods*. Six hundred and twelve randomly selected Nigerian adolescents aged 12 to 18 years were examined using the ICON in their school compounds. Descriptive statistics were employed in the data analysis. *Results*. Out of a total of 38.1% of the population found to need orthodontic treatment, there were more males and older adolescents. The overall mean ICON score for the population was 39.7 ± 25.3 SD with males having statistically higher mean ICON score. The grades of complexity of the population were 21.6% for very difficult and difficult, 7.5% moderate, and 70.9% mild/easy. *Conclusions*. Although just over a third of the adolescents were found to have a need for treatment, about a quarter of them were found to have difficult and very difficult complexity grades indicating a need for specialist care. The authors recommend the training of more specialist orthodontists in this region.

## 1. Introduction 

The assessment of orthodontic treatment need and complexity is necessary for the planning of orthodontic services in any given population, as well as training programmes for specialists [1]. Occlusal indices, such as the Index of Orthodontic Treatment Need (IOTN) [2] and the Dental Aesthetic Index (DAI) [3] have been used successfully around the world to provide information on orthodontic treatment need in various communities.

Richmond et al. [4] suggested that difficulty and complexity in orthodontics are synonymous and should be defined as a measurement of skill and effort while severity is a measurement of how far a malocclusion deviates from the normal. Meanwhile, Cassinelli et al. [5] also reported that complexity or difficulty is related to the severity of malocclusion and increases as the severity of the malocclusion increases. 

The Index of Complexity, Outcome, and Need (ICON) [6] which was developed based on the expert opinions of 97 practising orthodontic specialists from 9 countries has provided an internationally acceptable means of measuring orthodontic treatment need, complexity and outcome with a single measurement protocol. The index comprises five components: The Aesthetic Component of the IOTN (AC), amount of maxillary crowding or spacing, the presence or absence of crossbite, incisor open bite/overbite, and anteroposterior buccal relationship which are weighted as follows: AC (7), maxillary crowding or spacing (5), crossbite (5), incisor overbite/open bite (4), and anteroposterior buccal relationship (3). The components are measured, multiplied by their respective weights, and summed up to give an overall score. The cut-off point for treatment need is an ICON score of 43. Complexity values are graded from easy to very difficult, depending on the score obtained.

In Southwestern Nigeria, various studies have been carried out to assess the prevalence of malocclusion, though a large majority [7–14] were purely descriptive qualitative studies. Other studies on orthodontic treatment need have equally been done with the Index of Orthodontic Treatment Need (IOTN) and the Dental Aesthetic Index (DAI) [15–21]. Meanwhile the Index of Complexity, Outcome, and Need (ICON) has also been used to determine orthodontic treatment need and complexity both in the general population and in patients attending orthodontic clinics [22–24]. 

Generally, research, clinical practice, and specialist training in Southwestern Nigeria as it relates to orthodontics is much more advanced, as compared to Rivers State located in South-South Nigeria, where the specialty is relatively new. Outside the studies in Ibadan City of Nigeria, the authors search did not reveal any literature from other parts of Nigeria that assessed both orthodontic treatment need and complexity using the ICON. 

Moreover, ICON has been validated for the Nigerian population, shown to be useful in assessing orthodontic treatment need and complexity [22, 23] and to be effective for assessment of different facets of orthodontic provision, when compared with previously existing indices [25]. Therefore, the aim of this epidemiological study was to estimate the need for and the complexity of orthodontic treatment among 12 to 18 year-old schoolchildren in Rivers State, Nigeria, using the ICON. This is the first time such a study is being carried out in this part of Nigeria, and documentation of such important statistics will thus allow for informed planning of orthodontic services in this region.

## 2. Material and Methods

There are 23 Local Government Areas (LGAs) in Rivers State of Nigeria Due to security concerns at the time this study was being carried out; permission was granted by the Rivers State Ministry of Education for 12 LGAs, comprising 2 urban and 10 rural, out of which six were selected by ballot, consisting of one urban LGA (Port Harcourt) and five rural LGAs: Ikwerre, Omumma, Tai, Okrika, and Asari-Toru.

 Out of the list of schools obtained from the Rivers State Ministry of Education one school was selected by ballot from each of the six LGAs making a total of six schools. The students were randomly selected from each of the schools and the sample population consisting of 612 students with age range of 12 to 18 years was obtained comprising 299 (48.9%) males and 313 (51.1%) females. None of the students had undergone any form of orthodontic treatment.

The researcher was calibrated in the use of the ICON using dental casts by a senior colleague (COO) who is trained and experienced in the use of the index. An intraoral examination of the participating students was conducted by the researcher in the selected school compounds using natural illumination and strictly following the guidelines of the ICON. Disposable wooden spatulae and orthodontic millimetre rulers were used. The need for orthodontic treatment was defined as an ICON score of 43 and above while complexity was graded into easy (<29), mild (29–50), moderate (51–63), difficult (64–77), and very difficult (>77) in line with ICON guidelines.

### 2.1. Intraexaminer Reliability

Sixty-two of the students were selected randomly and reexamined by the researcher after a four-week interval. The two examinations were evaluated statistically.

The reproducibility of the ICON scores were assessed using Spearman's Rank Correlation Coefficient (*P* = 0.98), and excellent agreement was found. Intraexaminer consistency for the categorisation of treatment need into need and no need was expressed as the kappa reliability coefficient with a value of 0.93 indicating strong agreement, whilst the reliability of the complexity grades was also evaluated using W Kendall test with a value of 0.78.

### 2.2. Statistical Analysis

The data was analyzed statistically using the SPSS statistical package (Statistical Package for the Social Sciences Version 17.0 for Windows 2009, SPSS, Inc., Chicago, Ill, USA). 

### 2.3. Descriptive Statistics

The qualitative variable “gender” was described using frequencies and percentages. For the quantitative variable ICON score, mean for central tendency and standard deviation were used. For the ordinal variables, ICON categorization of treatment complexity, number (frequencies), and percentages were used for descriptive statistics. 

### 2.4. Inferential Statistics

Male and female subject differences with respect to ICON score were tested using Student *t*-test. To test for any dependence on gender of “complexity grades,” chi-square test was used.

## 3. Results

The mean age of the studied population was 15 ± 2.0 years; male was 14.9 ± 1.9 years and female was 15.0 ± 2.0. 

### 3.1. Orthodontic Treatment Need

About thirty-eight per cent (38.1%) of the studied population had a need for orthodontic treatment with a mean ICON score of 39.7 ± 25.3. There were statistically significant gender differences, the mean ICON score was higher in males (43.1 ± 26.3) than females (36.3 ± 23.8) (*P* = 0.001), and a higher number of males (43.5%) than females (32.9%) were found to be in need of orthodontic treatment (Table 1). Statistically significant age differences were also determined; twelve- and thirteen-year olds were less likely to have a treatment need (odd ratio = 0.58), while 17-year olds were found to be more likely to have need for orthodontic treatment (Odd ratio = 1.82) when compared with the other age groups (*P* = 0.04) (Table 2).

### 3.2. Orthodontic Treatment Complexity

**Figure 1:**
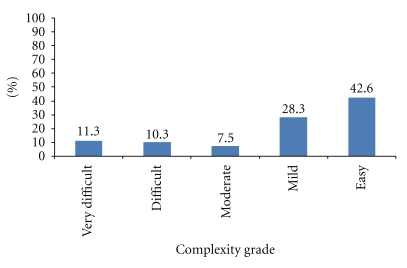
Complexity grades according to the Index of Complexity, Outcome, and Need.

The grades of orthodontic treatment complexity are shown in Figure 1. Easy complexity was found in 42.6% of the population, 28.3% had mild complexity, 7.5% moderate complexity, whilst 10.3% and 11.3% had difficult and very difficult grades of complexity, respectively. There were significant differences between complexity grades in males and females (*P* = 0.02) (Table 1), with twice as many males (15.1%) with very difficult complexity grades as females (7.7%) and more female (47.3%) than male students (37.8%) found to have easy categories of treatment need.

As the level of complexity of the malocclusion increased, a corresponding increase in treatment need was seen (Table 3). Of the students assessed to have a need for treatment (38.1%), none of them was found to have malocclusions of easy complexity while students without a treatment need did not have malocclusion that was categorized to be difficult or very difficult to treat. 

## 4. Discussion

The mean ICON score of 39.7 ± 25.3 obtained in this study was slightly lower than that obtained for a similar Nigerian population [24] of 12- to 18-year olds (41.93 ± 15.38). However, this study involved a larger sample size, and thus a wider range of occlusion was assessed. Likewise, the mean ICON score recorded in our study was also lower than the values obtained in prevalence studies on 12- and 13-year olds in Latvia (42.05) [26] and Senegal (42.31–44.46) [27] and 11–14 year-old Iranian schoolchildren (44.6 ± 24.83) [28]. These differences could be due to the wider age range of the population whilst racial variations may also be a factor.

Just over a third of the population in this epidemiological study was found to have a need for orthodontic treatment according to the ICON (38.1%). This is comparable to the value **(**35.3%) obtained for children in Latvia [26] but lower than 42% obtained for adolescents in Western Nigeria [24, 29], 44.1% in Senegal [27], and 46.6% reported among 11–14 year-old Iranian schoolchildren [28]. Studies conducted on orthodontic patients, however, with values of 82.1% in Nigeria [23], 94% and 86% in Greece [30] and United States of America [25, 31], respectively, have a much higher need for treatment than obtained in this study. This is expected because these studies were clinic-based and involve patients with recognised needs for treatment that brought them to the orthodontists. 

 The increase of orthodontic treatment need with age seen in this study is probably due to the fact that untreated malocclusion worsens with age as the permanent occlusion becomes established [32]. When assessed professionally, male adolescents had a significantly greater need for treatment than females which is consistent with the findings of Burden et al. [33], but inconsistent with the finding by Onyeaso [20] in a clinic-based study in Southwest Nigeria where clinical research has shown that more females recognise a need for orthodontic treatment than males [20]. These findings, however, are in contrast to other studies to assess orthodontic treatment need using the ICON, DAI, and IOTN conducted in Nigeria [15, 29, 34], Tanzania [35], Senegal [27], France [1], Kuwait [36], Latvia [26], and Iran [28] where there were no gender or age differences. 

Very difficult and difficult complexity grades of malocclusion accounted for almost a quarter of the adolescents (21.6%) which was similar to values obtained in Iranian schoolchildren (26%) [28] but in sharp contrast to that obtained among adolescents in Western Nigeria (9.9%) [24] and 10% in Latvia [26]. The majority of adolescents with malocclusion can be greatly reduced by intercepting and treating during childhood. In the Iranian study [28], only 1.1% of the studied population wore an orthodontic appliance, which indicates a dearth of interceptive orthodontics among Iranian children. Such is the case in Rivers State, where none of the children wore an appliance, and it is just recently that such services started at the University of Port Harcourt Teaching Hospital in the State. Much higher values were obtained in previous clinic-based studies in Nigeria [23], Greece [30], and United States [31] of 60.7%, 61% and 60%, respectively, due to the fact that these are orthodontic patients with obvious needs for treatment.

Moderately complex cases in this study were the least in number (7.5%) which is quite low compared with values from other prevalence studies, 16.1% in Nigeria [24], 14.1% in Latvia [26], and 15.1% in Iran [28]. This value is also much lower than that obtained in clinic-based studies carried out in Nigeria [23] (14.3%), Greece [30] (23%), and USA [31] (22%). 

Mild and easy cases in our study (70.9%) were found to be comparable to the 75% obtained in Western Nigeria [23] and 76% obtained in Latvia [26], higher than 58.5% amongst Iranian schoolchildren [28], but in contrast to values obtained among orthodontic patients in Nigeria [23] (25%), Greece [30] (16%), and USA [31] (18%).

Another finding in this study was the significant difference in complexity grades found between male and female adolescents. Twice as many male students (15.1%) had very difficult grades of complexity as compared with the female students (7.7%). This is similar to the findings in the recent Iranian study [28], where a greater number of male (14.8%) than female children (9.1%) were found to have very difficult grades of complexity of their malocclusions although this was not statistically significant (*P* > 0.05), unlike the present study.

In this study there were also highly significant associations between orthodontic treatment complexity and need because the higher the complexity grade, the higher the degree of treatment need and the greater the severity of the malocclusion. Similar findings have been documented in both epidemiological studies and in orthodontic patient populations in earlier studies in Ibadan [23, 24] using the ICON and DAI and in a US study [25]. The complexity of cases in a particular location is extremely important because cases classified as very difficult, difficult, and moderate in complexity require the skills of specialists to be adequately treated. Richmond et al. [4, 37], as well as Onyeaso and BeGole [31], found the pretreatment ICON score to be a good indicator of treatment difficulty. They reported that cases with higher pretreatment ICON scores took a longer time to treat. Cassinelli et al. [5] reported that complexity or difficulty in achieving an ideal occlusion increases as the severity of the initial malocclusion increases. 

## 5. Conclusion

The overall prevalence of orthodontic treatment need among adolescents aged 12–18 years in Rivers State, Nigeria was 38.1%. Most of the subjects fell into the easy grade of complexity while about a quarter was found to have difficult and very difficult grades of complexity.

As the level of complexity of malocclusion increased, a corresponding increase in treatment need was seen. Although no adequate explanation was found, orthodontic treatment need and complexity were found to be significantly higher amongst males and in the older age group. 

Considering the large percentages of adolescents found to have a need for orthodontic treatment as well as difficult and very difficult grades of treatment complexity, we recommend that more attention be given to the training of orthodontic specialists, and that orthodontic care should be subsidised by the Rivers State Government so that it can become affordable to the majority of these adolescents in need of such care. 

## Figures and Tables

**Table 1:** Assessment of orthodontic treatment need and complexity by gender according to the Index of Complexity, Outcome, and Need (ICON).

Variable	Frequency (%)		
	Male	Female	Total
Icon assessment score			
<43	169 (56.5)	210 (67.1)	379 (61.9)
≥43	130 (43.5)	103 (32.9)	233 (38.1)
Total	**299 (100)**	**313 (100)**	**612 (100)**
Mean	43.1 ± 26.3	36.3 ± 23.8	39.7 ± 25.3
Student's *t* statistic = 3.37, *P* = 0.001			

Complexity grade			
Very difficult	45 (15.1)	24 (7.7)	69 (11.3)
Difficult	33 (11.0)	30 (9.6)	63 (10.3)
Moderate	26 (8.7)	20 (6.4)	46 (7.5)
Mild	82 (27.4)	91 (29.1)	173 (28.3)
Easy	113 (37.8)	148 (47.3)	261 (42.6)
Total	**299 (100)**	**313 (100)**	**612 (100) **
*χ* ^2^ = 12.16, *df* = 4, *P* = 0.02			

**Table 2:** Relationship between age, gender, and assessed orthodontic treatment need according to the Index of Complexity, Outcome, and Need.

Variable	ICON assessment of need (%)		*χ* ^2^	Odd ratio	95% CI		*P*
	Need (*n* = 233)	No need (*n* = 379)			Lower	Upper	
Age (years)							
12	26 (28.0)	67 (72.0)	4.76	0.58	0.35	0.97	0.03*
13	22 (27.5)	58 (72.5)	4.36	0.58	0.33	1.00	0.04*
14	36 (38.7)	57 (61.3)	0.02	1.03	0.64	1.66	0.89
15	34 (45.3)	41 (54.7)	1.91	1.41	0.84	2.36	0.17
16	37 (37.4)	62 (62.6)	0.02	0.97	0.60	1.54	0.88
17	54 (50.0)	54 (50.0)	7.91	1.82	1.17	2.82	0.01*
18	24 (37.5)	40 (62.5)	0.01	0.97	0.55	1.71	0.92
Gender							
Male	130 (43.5)	169 (56.5)	7.25	1.57	1.11	2.21	0.01*
Female	103 (32.9)	210 (67.1)	7.25	0.64	0.45	0.90	0.01*

*Significant.

**Table 3:** Associations between orthodontic treatment need and complexity.

Assessment according to the ICON	Complexity grade					
	Very difficult	Difficult	Moderate	Mild	Easy	Total
Need	69 (29.6)	63 (27.0)	45 (19.3)	56 (24.0)	0 (0)	233
No need	0 (0)	0 (0)	1 (0.3)	117 (30.9)	261 (68.9)	379
Total	**69**	**63**	**46**	**173**	**261**	**612 **
*χ* ^2^ = 447.22, *df* = 4, *P* = 0.00.						
